# Calcium dynamics in small spaces: Lessons learned from modeling in dendritic spines

**DOI:** 10.1016/j.bpj.2025.09.038

**Published:** 2025-09-29

**Authors:** Kimberly J. McCabe, María Hernández Mesa, Padmini Rangamani

**Affiliations:** 1 Department of Computational Physiology, Simula Research Laboratory, Oslo, Norway; 2 Department of Mechanical and Aerospace Engineering, Jacobs School of Engineering, University of California, San Diego, San Diego, California; 3 Department of Pharmacology, School of Medicine, University of California, San Diego, San Diego, California

## Abstract

Spatiotemporal dynamics of calcium regulation in subcellular regions is critical for precise local control of cell signaling. Recent studies have shown that, in addition to biochemical control of localized calcium signaling through buffers and channels, the geometry of the small spaces in which calcium signaling occurs also matters. Geometric organization becomes particularly important when considering the role of organelles such as the mitochondria and endoplasmic reticulum in regulating calcium signaling. Here, we discuss recent advances in our understanding of calcium dynamics in small spaces such as dendritic spines and how computational modeling can reveal a complex interplay between geometry and receptor clustering. We close with other biological examples where such interactions may be important and suggest the possibility of generalizable biophysical principles of localized calcium control.

## Significance

Recent advances in imaging and reconstruction of 3D microscopy show that the geometries of neuronal structures are quite complex. Since cell shape is known to affect signaling pathways in many ways, it is natural to ask how the spatiotemporal dynamics of calcium, an important second messenger in neurons, are tightly regulated by geometric factors. Here, we discuss how computational modeling can give us insight into the role of subcellular architecture in dendritic spines. We also discuss the possibility that such geometric control might be relevant to other cell types such as cardiac myocytes and skeletal muscle where precise calcium control is critical for cellular function.

## Introduction

Neuronal communication occurs at synapses, which consist of three parts: a presynaptic terminal on one neuron, the postsynaptic terminal of another neuron, and the synaptic cleft between them (1). Dendritic spines are small protrusions (0.04–0.29 μm^3^) (2,3) along the dendrite of neurons and are the specific sites in neurons where most excitatory synapses are located (4). Though dendritic spines are relatively small subcellular compartments, they serve as hotbeds of biochemical activity (5). There are many comprehensive reviews on the different signaling and cytoskeletal remodeling events that occur in dendritic spines on different timescales (6,7,8). In this perspective, we focus on upstream calcium dynamics because it provides a framework upon which to explore the complexities of second messenger dynamics in small spaces.

Ca^2+^ ions are key to signal transduction in a wide range of biological processes. The interplay of Ca^2+^ with kinases, phosphatases, ion channels, exchangers, and transporters regulates a large number of cellular processes such as growth, differentiation, development, and gene expression. These cellular processes are critical in many cell types, and it is well known that Ca^2+^ plays a pivotal role in muscle contraction in both skeletal and cardiac muscle, synaptic communication in neurons, and secretion of hormones among other processes. Consequently, complex signaling systems involving Ca^2+^ kinetics are of great interest in cell biology and biophysics. Although advances in experimental techniques have allowed for a more comprehensive understanding of subcellular networks (9), many biological questions remain unanswered due to the complexity of biological systems. Computational and mathematical models are great tools that allow scientists to understand and analyze such systems with high accuracy, design new experiments, develop hypotheses based on simulations, predict the behavior of systems, and reduce the cost and amount of experimental procedures. Furthermore, computational modeling offers the possibility of integrating cellular signaling with the spatial organization and morphology of cells, which is also known to affect cellular function.

## Sources and sinks of Ca^2+^ in dendritic spines

**Figure 1:**
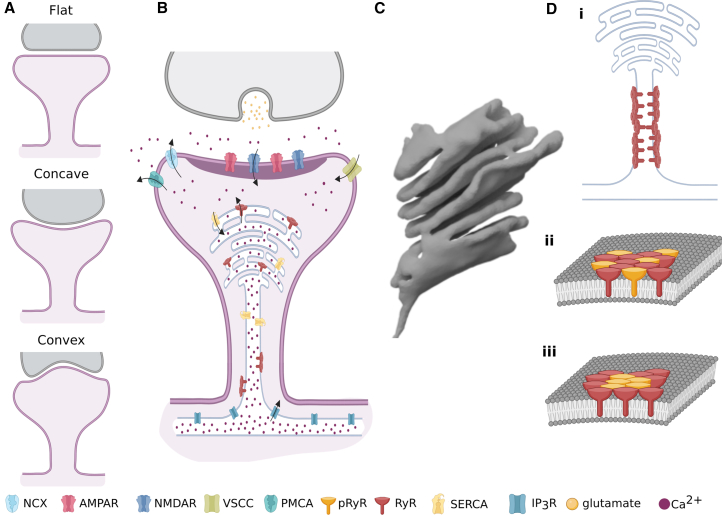
Schematic describing the small spaces in which Ca^2+^ signaling occurs in dendritic spines. (*A*) Synaptic cleft morphologies, (*B*) signaling between the plasma membrane and the spine apparatus (image from (1)), and (*C*) laminar structure of the spine apparatus. This spine apparatus was segmented and meshed by Lee et al. (11) using electron microscopy images from the mouse cerebral cortex originally imaged by Wu et al. (12). (*D*) Receptor clustering and phosphorylation patterns also affect Ca^2+^ dynamics; i: receptor clustering in the spine apparatus neck, ii: uniform distribution of phosphorylated (*yellow*) and non-phosphorylated (*red*) RyRs, and iii: concentration of phosphorylated RyRs toward the center of an RyR cluster.

The structure of the dendritic spine, which is separated from the dendritic branch of the neuron by a small neck, creates an isolated signaling compartment (10); see Fig. 1. Synaptic communication starts with a signal at the presynaptic terminal that leads to the release of a neurotransmitter such as glutamate into the synaptic cleft. After traversing the cleft, glutamate can activate glutamate N-methyl-D-aspartate (NMDA) receptors and *α* -amino-3-hydroxy-5-methyl-4-isoxazolepropionic acid (AMPA) receptors located in the postsynaptic density (PSD). The PSD is a specialized region of the postsynaptic membrane of dendritic spines characterized by increased protein density. Once activated by glutamate, NMDA receptors open and allow the influx of Ca^2+^ into the cytosol when paired with depolarization of the postsynaptic membrane to relieve a voltage-dependent block by magnesium ions (Mg^2+^) (13). AMPA receptors have relatively low permeability to Ca^2+^ ions (14). However, in some parts of the brain, this low flux can still impact Ca^2+^ kinetics (15,16). In addition, AMPA receptors compete with NMDA receptors for glutamate binding, which can further modulate NMDA receptor activation and Ca^2+^ influx (14,17).

Two other sources contribute to an increase in cytosolic Ca^2+^ in dendritic spines: influx through voltage-sensitive Ca^2+^ channels (VSCCs) and Ca^2+^ release from internal organelles (10,14). Plasma membrane Ca^2+^-ATPases (PMCAs) actively pump Ca^2+^ out of the cytosol, affecting the shape and duration of Ca^2+^ signals and maintaining Ca^2+^ homeostasis within the spine (18). During action potentials, the plasma membrane of dendritic spines is depolarized, activating VSCCs. This allows for an additional influx of Ca^2+^ ions into the cytosol. With an increase in Ca^2+^ from external sources, Ca^2+^ diffuses within the dendrites, initiating signaling cascades, and the processes for Ca^2+^ release from internal stores are activated. The main internal sources of Ca^2+^ ions are the mitochondria and the endoplasmic reticulum (ER). Only a subset of dendritic spines (around 48% of adult spines) contains smooth endoplasmic reticulum (SER) (3). Further, 10%–20% of adult spines display a specialized form of ER known as the spine apparatus (SA) (3,19,20). Jedlicka et al. demonstrated that the SA is important for the maintenance of late-phase long-term potentiation and is required for spatial learning, suggesting that spines containing this organelle are associated with more robust synaptic plasticity (19).

## Geometric considerations

The functional complexity of calcium signaling in excitable cells is highly dependent on unique morphological structures and spaces between two opposing membranes. The synaptic cleft is a small region with the presynaptic and postsynaptic membranes roughly 20 to 50 nm apart (21,22) (Fig. 1 *A*). The geometry of the synaptic cleft can modulate the diffusion of neurotransmitters released from the presynaptic terminal, impacting the probability of NMDAR activation (23). Synaptic morphology itself has been widely studied with respect to its role in synaptic plasticity (21,24). Changes in synaptic membrane morphology affect the geometry of the synaptic cleft (25) and can alter the spatial arrangement of NMDARs and AMPARs within the synaptic cleft (26). Furthermore, presynaptic membrane curvature is often associated with the efficiency of vesicle docking and neurotransmitter release (24,27,28). In an early study, Markus and Petit observed a shift from a balanced mix of concave and convex synapses in the early stages of development to predominantly convex synapses in mature adult rats (27). Medvedev et al. observed that synapses in mushroom spines became less concave (increasingly flat) after long-term potentiation, whereas thin spines showed very pronounced changes in PSD curvature, transforming from concave into convex (29). These changes align with the observations of Markus and Petit who found more convex spines in mature adult rats (27). Rusakov et al. investigated the role of glutamate diffusion within the synaptic cleft and tested the relationship between release distance from the cleft and NMDA/AMPA receptor activation (30). These studies indicate that the geometry of the synaptic cleft is important for modulating synaptic function.

Another important domain of interactions between two membranes is Ca^2+^ dynamics between the plasma membrane and the ER (Fig. 1 *B*). The SA is identified by its characteristic laminar structure of stacked discs (20) (Fig. 1 *C*). One must consider the intricate and dynamic morphologies of SAs in order to fully understand Ca^2+^ in this subcellular compartment (31). Cytosolic Ca^2+^ ions activate ryanodine receptors (RyRs), which allows for further Ca^2+^ release into the cytosol, a process known as Ca^2+^-induced Ca^2+^ release (CICR). Although IP_3_ is the main agonist of IP_3_ receptors (IP_3_Rs), some studies have suggested that IP_3_Rs also play a role in CICR (32,33). Thus, receptor organization on organelle membranes also plays an important role in overall Ca^2+^ handling.

Adding complexity to such membrane organization is the presence of organelle contact sites. 3D reconstructions of dendrites show that dendrites have an abundance of contact sites between the endoplasmic membrane and other membranes (12). Contact sites between the ER and the plasma membrane are shown to be periodically spaced at roughly 1 μm apart in rat hippocampal neurons and are thought to play an important role in calcium regulation (34).

## Experimental advances and limitations

Before describing computational approaches to modeling of Ca^2+^ in dendritic spines, it is important to acknowledge recent advances in experimental techniques that allow for improved computational exploration and to consider limitations that modelers should be aware of when making assumptions about subcellular geometries based on experimental imaging.

Small subcellular structures such as the dendritic spine could not historically be resolved morphologically by conventional light microscopy techniques because spine features are smaller than the diffraction limit (35). Recent advances in microscopy allow for nanoscale resolution, which can give modelers a better understanding of spine size and synapse properties. For example, stochastic optical reconstruction microscopy and photoactivated localization microscopy found an average spine neck diameter of 195 nm (36). A creative combination of cryo-electron tomography and cryo-correlative light and electron microscopy found that PSD thickness is approximately 20–50 nm (37). Super-resolution imaging has also been able to visualize specific receptor locations, for example noting the organization of AMPA receptors into stable nanodomains in neurons (38), and the clustering of RyRs in strategic locations in the cardiac dyad (39). Basnayake et al. were able to observe the distribution of sarcoplasmic/ER Ca^2+^-ATPase (SERCA) and RyRs within the SA using super-resolution stimulated emission depletion microscopy (40). Thus, advances in microscopy techniques at multiple length and time scales have allowed us to use experimental data to inform the questions that can be answered by biophysical modeling.

Although considering geometric data from experiments, sample preparation methods should also be considered by modelers when making geometric domains, meshes, or assumptions. For example, an illuminating comparison study of cryofixation versus traditional chemical fixation of tissue found that the spine neck is 30% thinner using a cryofixation approach, which is likely much closer to physiological conditions (41). In a small space such as the spine, this difference can have a large effect on diffusion and concentration gradients of ions in the compartment.

Beyond structural considerations, it is also important to rely on experimental data that can accurately measure dynamic cellular processes such as calcium handling. Higley and Sabatini provide a review of powerful methods for measuring calcium signals in dendritic spines, such as Ca^2+^-sensitive fluorophores, two-photon laser-scanning microscopy, and two-photon released caged neurotransmitters (10). Open challenges for drawing quantitative comparisons between models and experiments for time course data include parameter estimation and model formulation among others (42,43).

## Computational modeling approaches

When considering continuum approaches, biophysical modeling of Ca^2+^ in the cell can be performed using ordinary differential equations (ODEs), if the cell or subcompartments therein can be considered well-mixed, or partial differential equations (PDEs) if it is important to track concentrations over both space and time. In 2019, Ohadi et al. modeled the cAMP/PKA pathway in neurons using a well-mixed single-compartment ODE model with oscillatory calcium as an input and found that cAMP/PKA act as leaky integrators of calcium signals due to their nonreponsiveness to the high frequency component of Ca^2+^ oscillations (44). A 2023 paper on AMPAR dynamics in dendritic spines also used the well-mixed approach but considered two compartments (cytosol and plasma membrane) due to the consideration that AMPAR is a membrane-bound protein (45).

Given the spatial separation of sources and sinks, computational models that take realistic geometries into account have become an indispensable tool for investigating how Ca^2+^ dynamics evolve within small spaces (46,47). Since PDEs account for spatial dimensions, the boundaries of the simulated space must be defined. This enables simulations to consider meshes that mimic physiological morphology, whether these are theoretical idealized geometries or realistic geometries reconstructed from imaging techniques. Breit et al. showed that ER positioning and increased RyR and IP3R density compensate for difficulties in spine-to-dendrite communication present in spines with larger volumes due to limitations of diffusion (48). Ohadi et al. used idealized spine geometries to show that spatial localization of AC1 and PDE4 affects the sensitivity of the PKA/cAMP pathway to calcium inputs (49). A systematic study of calcium in various spine shapes and sizes showed that an optimal volume-to-surface area ratio of the spine can maximize calcium signaling in the spine, and indeed, it is a stronger predictor of calcium dynamics than size or shape alone (50). Integration of realistic geometries can provide further insights by investigating the way spine plasma membranes and SAs interact in real cells. Rosado et al. used TEM reconstruction to generate realistic spine geometries, confirming a positive correlation between PSD size and SA volume (51). They then used PDEs within the NeuroBox toolbox to find a critical RyR density in spines that could trigger “all or nothing” downstream calcium signals that depended on the ratio of SA neck width to spine neck width (51).

Recent advancements in microscopy imaging and meshing techniques (11) have enabled studies about the dynamics of Ca^2+^ signaling in reconstructed geometries derived from electron microscopy (13,11,52). In small spaces, stochasticity becomes an important factor in governing Ca^2+^ dynamics due to the small number of molecules present at any given time and the noise this causes (52,53,54). The stochastic nature of Ca^2+^ signaling in dendritic spines has been explored, for example in a study where system stochasticity was required to reproduce calcium bursts seen experimentally (55). The small volume of these structures, combined with the scarce number of Ca^2+^ channels and their stochastic behavior, has led several modeling groups to favor particle-based simulation. In 2005, Holcman used an idealized spine geometry and a set of stochastic equations to demonstrate that considering drift in addition to diffusion can explain slower apparent diffusion seen in experiments of calcium decay (56). They furthered this work in 2011 by specifically addressing the unique geometry of dendritic spines, which requires solving the “narrow escape problem” because of the long, narrow neck (57). Spatiotemporal dynamics of PKA in spines was investigated using similar methods, leading to the finding that colocalization of PKA and cAMP was important for increased PKA activity (58,59).

MCell (60,61) provides a robust framework for conducting particle-based stochastic simulations for biological processes and is particularly relevant for simulations of dendritic spines. Despite the computational complexity inherent in spatial simulations, the use of this simulation tool has shown insightful and accurate results related to the influence of morphology on signaling cascades in neurons (62,63,64). As a recent example, Friedhoff et al. used MCell in idealized spine geometries to posit that an IP3R model with nonlinear response to calcium binding and slow Ca^2+^-dependent inhibition most accurately reproduces calcium dynamics (65). In 2022, Bell et al. used MCell to simulate calcium dynamics in both idealized and realistic spine geometries, finding that higher spine volume/surface area ratio correlates with higher synaptic strength (52).

In some cases where increased spatial detail is only required in certain spatial domains, modelers might consider constructing hybrid models that solve, for example, membrane dynamics using ODEs, bulk cytosol dynamics using PDEs, and agent-based modeling in the spine region in order to maintain computational tractability while preserving stochasticity in crucial subcellular domains (66). Hybrid models hold the promise of offering a more comprehensive understanding of complex biophysical systems such as cardiac dyads, dendritic spines, and synapses, though coupling at interfaces can be difficult to implement. The STEPS simulator offers a promising framework, as it allows users to combine stochastic, reaction-diffusion, and well-mixed models (67,68). A comparison of the modeling approaches available, their use cases and assumptions, and some example software tools are listed in Table 1. We note that although this list is not comprehensive, it sets up a starting place for model development depending on the biological question being asked.

**Table 1:** Overview of commonly used computational modeling approaches and use cases

Modeling Framework	Key Assumptions/Properties	Primary Use cases	Software examples
Well-mixed ODEs	Spatially homogenous compartments Deterministic Ignores spatial gradients	Nonspatial signaling pathways	COPASI, MATLAB, VCell
Reaction-diffusion PDEs	Spatially continuousconcentrations Deterministic	Simulating concentration gradients in subcellular structures	VCell, COMSOL, NEURON, FEniCS
Particle based Stochastic (Agent-based)	Models discrete particles Includes stochastic noise Computationally expensive	Nanoscale domains with low molecule counts, single-channel gating	MCell, Smoldyn, STEPS
Electrodiffusion (PNP/KNP)	Couples ion diffusion with electric field effects Computationally expensive	Modeling ion dynamics in high-field regions	FEniCS, Finite element method (FEM) solvers
Hybrid models	Apply different methods to different spatial domains Requires careful coupling	Multiscale problems	STEPS, custom coded solutions

## Lessons learned from Ca^2+^ modeling in dendritic spines

Our previous work modeling stochastic small molecule dynamics in neurons has provided valuable insights into how dendritic spine geometry, synaptic geometry, and receptor localization influence receptor activation and calcium dynamics, highlighting key design principles for understanding synaptic and calcium signaling. One study demonstrated that synaptic geometry has a significant impact on NMDAR activation, with structural factors such as synaptic cleft width and membrane curvature playing crucial roles (23). Specifically, increasing the curvature of synaptic membranes can compensate for signal attenuation in larger cleft widths, and nonparallel membrane configurations can optimize receptor activation by increasing the surface area/volume ratio of the cleft, increasing glutamate residence time and reducing glutamate escape. Additionally, clustering of NMDARs improves receptor activation, emphasizing the importance of spatial receptor organization in synaptic function.

More recently, we focused on calcium dynamics in spines with and without an SA, revealing how the geometry of the spine membrane and the localization of RyRs and IP_3_Rs influence calcium transients (1) (Fig. 1 *D*i). An SA with RyRs localized in the neck region maximizes calcium signals in the dendrite, supporting spine-to-spine communication, whereas RyR localization at the spine head increases calcium in the spine but does not serve to shuttle calcium to the dendrite as effectively. This finding builds on previous stochastic modeling by Basnayake et al., which, when combined with experimental calcium uncaging studies, proposed that localizing RyRs at the SA base and SERCA at the head could efficiently amplify calcium signals to accelerate communication between the spine and dendrite (40). These findings illustrate how spine geometry and receptor placement can modulate calcium signaling, especially in the presence of SA, offering design principles for how cellular architecture affects neurotransmission and calcium dynamics. Collectively, these studies highlight the critical role of geometry and receptor distribution in regulating synaptic efficacy, plasticity, and calcium signaling, providing a deeper understanding of the interplay between structure and function in dendritic spines.

In other studies, we have explored how the presence of mitochondrial-ER contact sites can affect Ca^2+^ and ATP dynamics in dendrites using PDEs (69). However, more work is needed to combine realistic geometries and distributions of contact sites (such as the ones discovered in (34)) with particle-based modeling (61).

## Future outlook

### Considerations of electrodiffusion

Electrodiffusion is a phenomenon in which charged particles experience net flux due to concentration gradients combined with electrical potential gradients and is described by the Nernst-Planck equation. When investigating nanometer-scale domains, electrical fields certainly play a role in the movement of ions and should be considered when tractable. Indeed, electrodiffusive effects in dendritic spines have been predicted theoretically for decades (70). Including these dynamics in a computational model for Ca^2+^ dynamics requires solving the Poisson-Nernst-Planck (PNP) equations in three dimensions for ion electrodiffusion (71,72) and is thus highly computationally expensive. For more detailed information on electrodiffusion phenomena in neurons, we refer the reader to a review article on the matter by Savtchenko et al. (73).

Recent advancements have led to the development of mathematical frameworks aimed at studying and simplifying calculations related to subcellular electrodiffusion. These models involve solving the PNP equation, which combines the Nernst-Planck equation describing ion flux with the Poisson equation describing the electric field generated by these ions (74). A 2019 study using the PNP approach in dendritic spines found that, indeed, electrodiffusion and spine geometry both play significant roles in excitatory dynamics, and excitation effectively decreases resistance in the spine, facilitating synaptic strength (75). Boahen et al. have shown that electrodiffusion is an important consideration when modeling ion movement in spines due to the small and irregular geometries and the presence of an electric field near the membrane (76).

In the last decade, advancements have been made toward tractability of this system by simplifying the PNP equation to assume bulk electroneutrality, leading to the Kirchoff-Nernst-Planck (KNP) equation (77). A similar approach has also been applied to a larger system using a geometry of nine adjacent neurons and modeling the extracellular, membrane, and intracellular spaces using the KNP model (also known as the KNP-EMI model), demonstrating the scalability of this approach into tissue through simplification of the underlying electrodiffusion equations (78). Recent work with KNP-EMI has helped to improve the tractability of the model by introducing scalable solvers that can be applied to even more complex and realistic geometries (79). The above approaches all require the use of PDEs and can thus become computationally complex and expensive, especially in larger, more detailed geometries, so the introduction of open-source scalable solvers like this one are welcome.

### Beyond the neuron: Ca^2+^ modeling in other excitable cells

The principles of studying Ca^2+^ dynamics in small spaces extend beyond dendritic spines and neurons. Initial studies focusing on simulating Ca^2+^ dynamics in cardiac dyads date back to the 1990s. In 1992, Peskoff et al. developed a 1D deterministic model to simulate the effect of Ca^2+^ binding to the plasma membrane in the cardiac dyad (80). This model was later adapted to include Ca^2+^ flux through L-type calcium channels and the sodium-calcium exchanger (81). Soon after, a stochastic model of the RyR model was developed to study the conditions under which Ca^2+^ flux through the L-type calcium channels can activate Ca^2+^ release from the RyRs (82).

Using stochastic RyR simulations, Sobie et al. (83) investigated the effect of the number of RyRs on Ca^2+^ sparks and how these terminate. Utilizing spatial stochastic simulations with MCell and idealized geometries, Koh et al. (84) demonstrated that the cleft height has a larger impact on Ca^2+^ sparks than the cleft width. They also studied how many dyads are needed to obtain noise-free Ca^2+^ signals. Moreover, Tanskanen et al. (85) examined how electrodiffusion affects the movement of individual Ca^2+^ ions, highlighting the need for stochasticity when modeling Ca^2+^ diffusion in the dyad due to its small volume. With the publications from Koh et al. (84) and Tanskanen et al. (85), we observe that particle-based spatial stochastic models have been of interest in calcium release unit modeling for over 15 years.

With respect to spatial simulations with reconstructed geometries, Hake et al. (86) developed tools to generate geometrical meshes from electron tomographic images. This allowed them to model Ca^2+^ diffusion within the sarcoplasmic reticulum and the cytosol to investigate the effects of the sodium-calcium exchanger, SERCA, and calsequestrin localization on Ca^2+^ sparks. Kolstad et al. combined 3D dSTORM imaging with stochastic computational modeling investigate Ca^2+^ release through clusters of RyRs, finding that dispersion of RyRs in heart failure decreases the incidence of Ca^2+^ sparks (87) (Fig. 1 *D*ii, iii). A subsequent study investigated the experimental finding that RyR phosphorylation moves toward the center of clusters in heart failure (88). Using similar stochastic modeling methods as the Kolstad study, the modelers concluded that phosphorylation toward the center of an RyR cluster increases the probability of Ca^2+^ spark generation across a series of increasingly dispersed clusters, perhaps acting as a compensatory mechanism to the RyR dispersion observed in heart failure (89).

Advances in high-resolution imaging have allowed for even more detailed investigation of Ca^2+^ movement and interactions in realistic geometries with multiple organelles. Colman et al. developed a whole-cell model with realistic geometries of Ca^2+^ release including sarcoplasmic reticulum, T-tubules, and dyadic volumes to reproduce pro-arrhythmic electrophysiological environments observed experimentally (90). A recent work by Hirakis et al. used discrete stochastic modeling of the dyad to demonstrate the delicate interplay between geometry and receptor localization/activation required to induce calcium-induced calcium release, demonstrating the degree to which CICR is dependent on the integrity of the calcium release unit (91). These increasingly detailed examples in the cardiac dyad demonstrate the utility of biophysically detailed models of subcellular calcium handling and the universality of structure-function relationships across cell types.

Although these studies represent a different cell type, many organelles and messengers are preserved between neurons and muscle cells. Both cell types are excitatory, depend on Ca^2+^ to carry information spatially within the cell, and rely on small-volume subcompartments to restrict diffusion and amplify signals. Therefore, modelers can learn many lessons from the techniques employed in both cell types to make decisions about how to approach simulation questions related to calcium and other second messengers in small cellular spaces.

### Limitations and next steps for other biological contexts and questions

A key limitation in modeling calcium dynamics in dendritic spines is the accurate parameterization of the models. The main challenge in parameterizations is given by experimental constraints and biological variability. Several mathematical approaches have been proposed for quantifying the amount of uncertainty and estimate parameters considering the lack of biological experiments (42,92,93). As an example, modelers often assume a constant diffusion coefficient for calcium diffusion, although this value can be significantly affected by local factors such as membrane geometry, restricted mobility, and the presence of Ca^2+^ buffers (94,95). Buffering, in particular, introduces substantial complexity, with both the buffer concentration and the binding kinetics needing to be estimated. These parameters are frequently adjusted in models, often in an ad hoc manner to reproduce experimental calcium transients. However, these values are difficult to measure directly in biological experiments and can vary significantly between cell types. In general, modelers often choose buffer concentrations and kinetics based on in vitro experiments or previous computational models, which may not realistically reflect the in vivo behavior (96). Other parameters, such as receptor densities and ion channel kinetics, present similar challenges: receptor distributions are typically measured at the whole-cell level despite subcellular clustering, and although single-channel kinetics are well-characterized, the functional consequences of channel clustering are harder to measure experimentally. Moving forward, combining advanced imaging techniques, biochemical assays, high-resolution measurements, and computational biophysical models will allow for more accurate estimation of these parameters and improve the predictive power and biological relevance of computational models of calcium signaling in dendritic spines.

Not only is Ca^2+^-mediated signaling affected by compartmental geometry, but signaling also affects the geometry by reorganizing cytoskeletal architecture in synaptic plasticity (97,98,99). Therefore, there is a need for models that include feedback between signaling and structural changes to the cell geometry. Modelers have developed mathematical frameworks for understanding cytoskeletal elements such as actin assembly in spines (98,99,100,101,102), but more work is needed to integrate feedback explicitly by coupling signaling to actin assembly and developing moving boundary frameworks for subcellular spatial models. We discussed this topic in some detail in (103).

Small subcellular compartments and localization of sources and sinks are not limited to Ca^2+^ and are not limited to dendritic spines. Modelers can take lessons from dendritic spine modeling and use these frameworks in a variety of analogous subcellular and cellular systems. We have provided extensive examples in cardiac cells in the section above, but these principles can also be used in other organelles and subcellular compartments present in other cell types. For example, ODE models have been used to investigate transmembrane currents and Ca^2+^ activity in cilia (104). Systems of nonlinear equations have simulated binding, recycling, and internalization of extracellular vesicles (105). ODE and stochastic modeling techniques have been combined into a hybrid framework to provide insights into mechanisms of rear retraction in migrating cells (106). Beyond Ca^2+^, similar modeling techniques and assumptions can be applied to other molecules and second messengers, which are reviewed elsewhere; examples include cAMP and PKA (107), IP3 (108), and CaMKII (109,110). As described throughout the text, computational modeling has been a valuable tool for understanding complex questions in dendritic spines such as the reasoning behind receptor clustering, the role of dendritic spines in efficient signaling within neurons, and the effect of compartmental geometry on Ca^2+^ signaling and diffusion. It is our hope that this review of computational methods for investigating Ca^2+^ signaling in dendritic spines can indeed be generalizable to open questions in the field by identifying the types of questions that can be investigated using these models and providing insights for the broader computational physiology community.

## Acknowledgments

M.H.M. and K.J.M. are supported by the Simula-UCSD-University of Oslo Research and PhD training (SUURPh) program, an international collaboration in computational biology and medicine funded by the 10.13039/501100017488Norwegian Ministry of Education and Research. This work was supported in part by 10.13039/100000181Air Force Office of Scientific Research (AFOSR) MURI FA9550-18-1-0051 to P.R.

## Author contributions

All three authors drafted, edited, and finalized the manuscript text.

## Declaration of interests

P.R. is a consultant for Simula Research Laboratories in Oslo, Norway, and receives income. The terms of this arrangement have been reviewed and approved by the University of California, San Diego, in accordance with its conflict of interest policies.
